# Phase I Trial of Intra-arterial Administration of Autologous Bone Marrow-Derived Mesenchymal Stem Cells in Patients with Multiple System Atrophy

**DOI:** 10.1155/2021/9886877

**Published:** 2021-10-19

**Authors:** Seok Jong Chung, Tae Yong Lee, Yang Hyun Lee, KyoungWon Baik, Jin Ho Jung, Han Soo Yoo, Chang Jae Shim, Hyojin Eom, Ji-Yeon Hong, Dong Joon Kim, Young H. Sohn, Phil Hyu Lee

**Affiliations:** ^1^Department of Neurology, Yonsei University College of Medicine, Seoul 03722, Republic of Korea; ^2^Department of Neurology, Yongin Severance Hospital, Yonsei University Health System, Yongin 16995, Republic of Korea; ^3^Bioengineering Institute, CORESTEM Inc., Seoul 04763, Republic of Korea; ^4^Department of Neurology, Busan Paik Hospital, Inje University College of Medicine, Busan 47392, Republic of Korea; ^5^Department of Radiology, Yonsei University College of Medicine, Seoul 03722, Republic of Korea; ^6^Severance Biomedical Science Institute, Yonsei University College of Medicine, Seoul 03722, Republic of Korea

## Abstract

**Background:**

This study is aimed at investigating the safety and tolerability of the intra-arterial administration of autologous bone marrow-derived mesenchymal stem cells (BM-MSCs) in patients with multiple system atrophy- (MSA-) cerebellar type (MSA-C).

**Methods:**

This was a single-center, open-label phase I clinical trial in patients with MSA-C. A three-stage dose escalation scheme (low-dose, 3.0 × 10^5^ cells/kg; medium-dose, 6.0 × 10^5^ cells/kg; high-dose, 9.0 × 10^5^ cells/kg) was applied to determine the maximum tolerated dose of intra-arterial administration of BM-MSCs based on the no-observed-adverse-effect level derived from the toxicity study. The occurrence of adverse events was evaluated 1 day before and 1, 14, and 28 days after BM-MSC therapy. Additionally, we assessed changes in the Unified MSA Rating Scale (UMSARS) score 3 months after BM-MSC treatment.

**Results:**

One serious adverse drug reaction (ADR) of leptomeningeal enhancement following the intra-arterial BM-MSC administration occurred in one patient in the low-dose group. The safety review of the Internal Monitoring Committee interpreted this as radiological evidence of the blood-brain barrier permeability for MSCs. No other ADRs were observed in the medium- or high-dose groups. In particular, no ischemic lesions on diffusion-weighted images were observed in any of the study participants. Additionally, the medium- and high-dose groups tended to show a slower increase in UMSARS scores than the low-dose group during the 3-month follow-up.

**Conclusion:**

The present study confirmed that a single intra-arterial administration of autologous BM-MSCs is a safe and promising neuroprotective strategy in patients with MSA-C.

## 1. Introduction

Multiple system atrophy (MSA) is a sporadic neurodegenerative disorder that presents rapid disease progression and fatal prognosis. The pathological hallmark of MSA is *α*-synuclein-immunoreactive glial cytoplasmic inclusions in many regions [1], and the inclusions appear to have a strain of *α*-synuclein that is biologically distinct from that of other *α*-synucleinopathies (i.e., Lewy body disease) [2]. The pathogenesis of MSA is not yet fully understood, although some pathways have been proposed, including axonal degeneration with *α*-synuclein deposition, mitochondrial dysfunction, microgliosis, and oxidative stress [3]. Additionally, it remains uncertain whether the *α*-synuclein aggregation in MSA is a direct cause of neurodegeneration or a mere byproduct of other pathogenic pathways [4].

Accordingly, to slow disease progression of MSA, a multitarget therapeutic strategy that modulates the overall neurodegenerative process would be more promising than a single-target or protein-based approach. Several therapeutic candidates have failed to modify the disease course of MSA, including growth hormone [5], riluzole [6], minocycline [7], lithium [8], rifampicin [9], rasagiline [10], and epigallocathecin gallate [11]. Only the administration of autologous mesenchymal stem cells (MSCs) via the intra-arterial/intravenous [12, 13] or intrathecal routes [14] have shown any effect. Indeed, MSCs secrete various cytokines and growth factors that exert neuroprotective effects [15] and directly modulate *α*-synuclein-related neurodegeneration [16–19]. As such, MSCs may be the most suitable candidate for MSA treatment.

In previous investigator-initiated trials, we demonstrated the safety and clinical efficacy of intra-arterial and intravenous administration of autologous bone marrow-derived MSCs (BM-MSCs) in patients with MSA-cerebellar type (MSA-C) [12, 13]. In the present study, we conducted a single-center, phase I, dose-escalation sponsor-initiated trial to investigate the safety and tolerability of a single intra-arterial injection of autologous BM-MSCs in patients with MSA-C and to obtain approval from the Korean Ministry of Food and Drug Safety for this therapeutic strategy that is available commercially in the future [20].

## 2. Materials and Methods

### 2.1. Study Design

The present study was an open-label, phase I clinical trial of intra-arterial administration of autologous BM-MSC in patients with MSA-C at Severance Hospital, Yonsei University Health System, Seoul, South Korea. A three-stage dose escalation scheme was applied to determine the maximum tolerated dose of BM-MSC based on the no-observed-adverse-effect level (NOAEL) derived from the toxicity study: the low-dose group (*n* = 3) received a single dose of 3.0 × 10^5^ cells/kg, the medium-dose group (*n* = 3) received a single dose of 6.0 × 10^5^ cells/kg, and the high-dose group (*n* = 3) received a single dose of 9.0 × 10^5^ cells/kg, which was the highest dose for which we wanted to confirm safety based on our preclinical data (Supplementary Methods). Patients were advanced to the next dose group if all three patients were safely injected; if a serious adverse drug reaction (ADR) occurred in one of the three patients, an additional three patients were planned to be enrolled to confirm the safety and tolerability of that dose. We determined the sample size with a relatively small number considering that the safety of MSC treatment has been reported in our previous investigator-initiated trials [12, 13].

### 2.2. Participants

Patients who had been clinically diagnosed with probable MSA-C [21] were eligible for this study. To ensure that patients with relatively early-stage MSA were enrolled, the following inclusion criteria were implemented: (1) total Unified MSA Rating Scale (UMSARS) score between 30 and 50 and (2) disease duration of <5 years since MS diagnosis. The other inclusion criteria were (1) age between 30 and 75 years; (2) supportive findings from structural and/or functional imaging studies at the time of diagnosis (i.e., cerebellar atrophy on brain magnetic resonance imaging (MRI) or decreased glucose metabolism in the cerebellum on ^18^F-fluorodeoxyglucose positron emission tomography [PET]); and (3) no evidence of hematological abnormalities or suspected bone marrow failure. The exclusion criteria were (1) Korean version of the minimental state examination (K-MMSE) [22] score less than 24; (2) diagnosis of dementia according to the DSM-IV criteria; and (3) severe white matter hyperintensities or cerebrovascular lesions on brain MRI; (4) mutations in spinocerebellar ataxia type 1, 2, 3, 6, 7, 8, and 17 genes; (5) other major neurological disorders (e.g., stroke or brain surgery); (6) severe medical comorbidities (e.g., hepatic, renal, or heart failure, uncontrolled bleeding tendency, or malignancy) or febrile condition; (7) active infection with hepatitis B virus, hepatitis C virus, human immunodeficiency virus, cytomegalovirus, human T-cell lymphotropic virus type I/II, or syphilis; and (8) use of drugs that likely affect bone marrow function (e.g., heparin, immunosuppressive drugs such as steroid, and antibiotics such as cefazolin and rifampicin). All patients with MSA-C who visited the outpatient clinic between May 2018 and September 2019 were screened according to these eligibility criteria.

**Figure 1:**
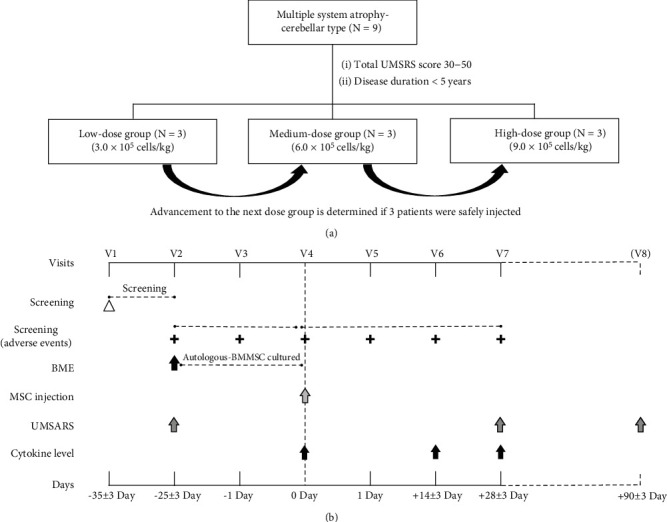
Study flow diagram. (a) Nine patients with multiple system atrophy-cerebellar type were enrolled in the present study. A three-stage dose escalation scheme was applied to determine the maximum tolerated dose of BM-MSCs. (b) According to the protocols of the clinical trial, patients visited the clinic seven times (V1–V7) to assess the safety and tolerability of intra-arterial administration of BM-MSCs. To explore the clinical efficacy, additional clinic visits were made 3 months after the intra-arterial administration (V8). Abbreviations: UMSARS: Unified Multiple System Atrophy Rating Scale; BME: bone marrow extraction; MSC: mesenchymal stem cell; BM-MSC: bone marrow-derived mesenchymal stem cell.

The study consisted of a screening period of 35 days, as well as a treatment and follow-up period of 28 days (Figure 1). During the 35-day screening period (visits 1–3 [V1–V3], day -35 to day -1), patients visited the clinic to determine their eligibility at V1. If enrolled, they underwent bone marrow extraction at V2. After harvesting of BM-MSCs from 50 to 70 mL of bone marrow aspirate, patients visited the clinic 1 day before the intra-arterial injection to assess the safety and tolerability at V3. Subsequently, patients received a single injection of intra-arterial autologous BM-MSCs at visit 4 (V4) and were followed up to assess the safety and tolerability of this procedure (visits 5–7 [V5–V7]; day 1 to day 28 after the intra-arterial BM-MSC injection).

### 2.3. Standard Protocol Approvals, Registrations, and Patient Consents

This study was approved by the Institutional Review Board of Yonsei University Severance Hospital (4-2017-0969) and by the Korean Ministry of Food and Drug Safety (30912). Written informed consent was obtained from all participants. The trial was registered at http://www.clinicaltrials.gov (identifier: NCT03265444).

### 2.4. MSC Preparation

The MSCs (CS10BR05) were isolated, expanded, and analyzed under good manufacturing practice conditions at CORESTEM Inc. (Seoul, South Korea), based on the guidelines of the International Society for Cellular Therapy. Bone marrow mononuclear cells were isolated using Ficoll density gradient centrifugation (Ficoll-Paque™ Premium; GE Healthcare Bio-Sciences AB, Uppsala, Sweden). The mononuclear cells (2 × 10^5^ cells) were placed in an 175 cm^2^ flask (Thermo Scientific Nunc, Roskilde, Denmark) and cultured in CSBM-A06 medium (CORESTEM Inc., Seoul, South Korea) containing 10% fetal bovine serum (Life Technologies Corp., Grand Island, NY, USA), 2.5 mM L-alanyl-L-glutamine (Biochrom AG, Berlin, Germany), and 1% penicillin–streptomycin (Biochrom AG, Berlin, Germany) in a humidified incubator at 37°C with 7% CO_2_. The nonadherent cells were removed after the initial plating, and the medium was replaced twice a week. Cells were harvested at 80% confluency using 0.125% trypsin-EDTA (Life Technologies Corp., Grand Island, NY, USA) and subcultured. Every harvest of MSCs was characterized by phenotypic analysis of the cell surface antigens. Using flow cytometry, the MSCs were shown to be negative for the hematopoietic markers CD34 and CD45 and positive for a number of adhesion molecules, including CD29, CD44, CD73, CD90, and CD105 (Table S1). To confirm sterility, samples were cultured against bacteria, fungi, viruses, and mycoplasma, and real-time polymerase chain reaction was performed to detect mycoplasma contamination. One further bone marrow aspiration and harvest of MSCs were allowed in cases of inadequate cell growth.

### 2.5. Intra-arterial Administration of BM-MSC

Patients were lying in the supine position on an examination table, and percutaneous access was obtained via the right femoral artery. A catheter was advanced into the cervical portion of bilateral internal carotid arteries (ICAs) and the proximal portion of dominant vertebral artery (VA). The assigned doses of the autologous BM-MSCs (CS10BR05) were then injected through the catheter into these three parts, as previously described [13]: 1/4 volume in each ICA and 1/2 volume in the dominant VA. For example, in the low-dose group, 0.75 × 10^5^ cells/kg were injected into each ICA and 1.5 × 10^5^ cells/kg into the dominant VA.

### 2.6. Assessment of Safety and Tolerability of Intra-arterial Injection of BM-MSC

Baseline assessment prior to intra-arterial BM-MSC administration included a history taking of general medical conditions, physical examination, vital signs, laboratory tests, chest X-ray, electrocardiogram, whole-body PET (to exclude malignancy), K-MMSE (to exclude dementia), and UMSARS.

To observe any acute adverse events (AEs) of BM-MSC administration, vital sign monitoring and neurologic/physical examination were performed 60 minutes before injection and 60 minutes, 120 minutes, and 240 minutes after injection. To evaluate any pathological findings associated with the injection (e.g., newly developed ischemic lesions), all participants underwent a brain MRI scan 1 day after the procedure (V5). Participants visited the outpatient clinic twice every 2 weeks after the procedure (i.e., V6–V7) for recording of AEs; at these visits, dose-limiting toxicity (DLT), physical examination, vital signs, and laboratory tests were recorded.

### 2.7. Secondary Outcomes

#### 2.7.1. Clinical Efficacy: Longitudinal Changes in UMSARS Scores

To explore the efficacy of MSC therapy, we scheduled an additional clinic visit 3 months (V8) after the intra-arterial administration of BM-MSCs (Figure 1). The severity of neurological deficits was assessed using the UMSARS score (part I, a historical review of disease-related impairments; part II, motor examination; and part IV, global disability scale) at additional visits.

#### 2.7.2. Analysis of Mechanism: Cytokine Bio-Plex Assay and Neurofilament Light Chain (NF-L) Immunoassay

To analyze the mechanism, plasma samples were obtained at scheduled visits (V4, V6, and V7) after intra-arterial administration of BM-MSCs. The plasma levels of 27 cytokines were selected and measured using the Bio-Plex200 Cytokine Assay System (Bio-Rad Laboratories, Hercules, CA, USA; see Supplementary Methods). Changes in plasma NF-L level were also assessed using enzyme-linked immunosorbent assay (ELISA) (ready-to-use sandwich ELISA kit [LS-F6701]; LSBio, Seattle, WA; Supplementary Methods).

### 2.8. Statistical Analysis

The frequency of AEs was expressed as number (percentage). Repeated-measure analysis of variance (ANOVA) was used to assess the longitudinal changes in UMSARS scores from baseline to 3 months after MSC injection. To assess the clinical efficacy of intra-arterial MSC injection, we compared longitudinal changes in UMSARS scores (part I, part II, and total) between the low-dose group (*n* = 3) and the medium- and high-dose groups (*n* = 6). Plasma levels of cytokines and NF-L at scheduled visits after MSC treatment (V6 and V7) were compared with those at baseline (V4) in MSC treatment groups using the Wilcoxon signed-rank test. Statistical analyses were performed using SPSS software (version 25.0; IBM Corp., Armonk, NY, USA). Results with a two-tailed *p* < 0.05 were considered statistically significant.

## 3. Results

### 3.1. Participants and Treatment

Nine patients (5 men and 4 women) who had been diagnosed with MSA-C <5 years prior were screened and enrolled in the present trial. All patients met the enrollment criteria, and none declined participation before commencement. According to the dose escalation scheme, three patients were consecutively assigned to each dosage group.

The demographic characteristics of study participants are listed in Table 1. The median age and disease duration of the nine patients were 57 years (minimum, 48 years; maximum, 68 years) and 30.1 months (minimum, 11.1 months; maximum, 44.7 months), respectively. The median total UMSARS score at screening (V1) was 40 (minimum, 30; maximum, 46). No medical history or disorders that could influence the study were reported, although one patient (S-103) had a history of hypertension and dyslipidemia; none of the other patients had any notable medical history. All patients completed the planned seven visits, as well as the additional follow-up visit 3 months after MSC administration.

### 3.2. Safety and Tolerability

**Figure 2:**
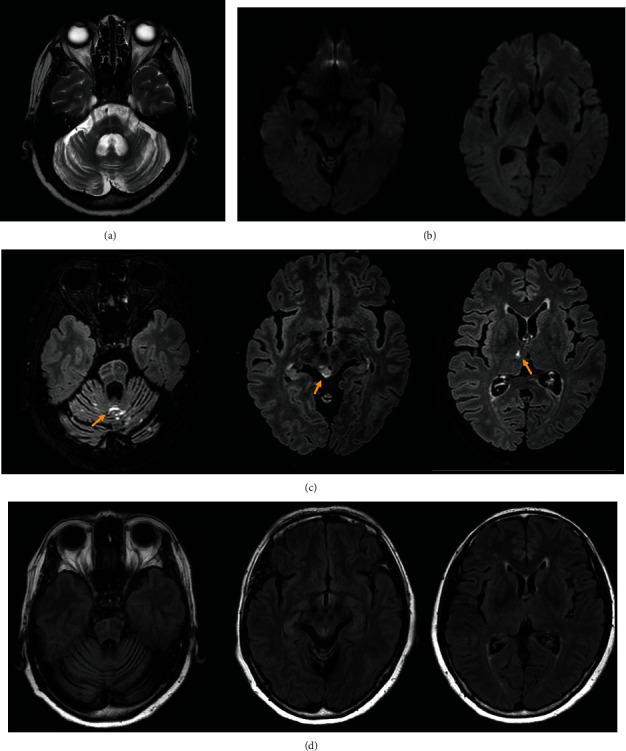
Brain magnetic resonance imaging (MRI) findings in patient S-103. (a) T2-weighted images. The brain MRI revealed findings consistent with multiple system atrophy including atrophy in the pons, middle cerebellar peduncle, and cerebellum. (b) Diffusion-weighted images on the day following the intra-arterial mesenchymal stem cells. Acute ischemic lesions were not observed. (c) Fluid-attenuated inversion recovery (FLAIR) with enhancement images on the day following the intra-arterial mesenchymal stem cells. Leptomeningeal enhancement occurred in the cerebellar folia along with enhancement in the right superior colliculus and right thalamus (yellow arrows). (d) FLAIR images four days after the procedure. The follow-up brain MRI demonstrated resolved hyperintensity in the right superior colliculus and right thalamus.

All nine participants successfully completed the clinical trial from the first enrollment (V1) to V7. A total of 15 AEs were reported in this period in five out of the nine patients (Table 2). Among them, two serious adverse events (SAEs) were observed in two patients in the low-dose group: (1) patient S-102 suffered from a urinary tract infection, which may not have been related to the MSC treatment; (2) patient S-103 showed leptomeningeal enhancement in the cerebellar folia along with enhancement in the right superior colliculus and right thalamus, on the brain MRI which performed 1 day after the intra-arterial BM-MSC administration according to the established protocols of the clinical trial (Figures 2(a)–2(c)). The patient did not develop any new neurological deficits during additional hospitalization. Four days after the procedure, the follow-up brain MRI demonstrated resolved hyperintensity in the right superior colliculus and right thalamus on fluid-attenuated inversion recovery (FLAIR) images (Figure 2(d)). The patient was discharged 5 days after MSC administration with no other AEs. Considering that the FLAIR image signal changes were reversible and that no additional neurological deficits occurred, the Data and Safety Monitoring Board of the study interpreted these MRI findings as radiological evidence of blood-brain barrier (BBB) breakdown with increased permeability. Therefore, the severity of the Suspected Unexpected Serious Adverse Reaction (SUSAR) was graded as moderate; the reaction was not considered an ADR corresponding to DLT. No other procedure- or MSC-related SAEs were observed in the study participants. In particular, no hyperintensity lesions were observed on the diffusion-weighted images of any study participants. The other 13 AEs were mild in severity (see Table 2), and in particular, no AEs were observed in the medium-dose group.

### 3.3. Longitudinal Changes in UMSARS Score

Table S2 shows the longitudinal changes in the UMSARS scores of the study participants from baseline to 3 months after intra-arterial administration of BM-MSC. Compared to the low-dose group, the medium- and high-dose groups appeared to have a slower rate of increase in UMSARS Part II score (group × time, *p* = 0.131) and total UMSARS score (group × time, *p* = 0.096), which did not reach statistical significance because each treatment group contained only a small number of patients (Table 3).

### 3.4. Changes in the Plasma Levels of Cytokines and NF-L

Among 27 cytokines, 17 (63.0%) were detectable at the baseline assessment (V4), and their plasma levels were not significantly changed at the 14-day (V6) and 28-day (V7) visits in any of the treatment groups. However, there was a trend toward decreases in the concentrations of proinflammatory cytokines, including interleukin-1*β* (IL-1*β*), tumor necrosis factor-*α* (TNF-*α*), and monocyte chemoattractant protein-1 (MCP-1) at the 14-day (V6) and 28-day (V7) visits in the medium- and high-dose groups, although these changes did not reach statistical significance. Additionally, the plasma levels of NF-L were not changed at the 1-month follow-up in the medium- and high-dose groups, while there was a trend toward increases in plasma NF-L levels in the low-dose group (Table S3 and Figure S1).

## 4. Discussion

In this phase I clinical trial of autologous BM-MSCs, which use a three-stage dose escalation scheme, we confirmed the safety and tolerability of the intra-arterial administration of BM-MSCs at a single dose of 9.0 × 10^5^ cells/kg in patients with MSA-C. Although we did not observe significantly favorable motor outcomes because the sample size was small, the medium- and high-dose groups tended to have a slow rate of increase in USMARS score. Additionally, the plasma levels of some proinflammatory cytokines tended to be decreased after BM-MSC administration in the medium- and high-dose groups. These findings suggest that intra-arterial administration of autologous BM-MSCs is a safe and promising neuroprotective strategy in patients with MSA-C. Further studies with a larger sample size and multiple administration schedules are required to confirm the clinical efficacy of MSC treatment.

MSA is a rapidly progressive and fatal neurodegenerative disorder. Although no therapies have been proven to cure the disease or slow its progression [23], a growing body of evidence has suggested that cell therapy using MSCs is a promising therapeutic strategy for MSA [12–14, 24]. It is well established that MSCs can migrate to injury sites and secrete various neurotrophic factors that exert neuroprotective effects [15, 25], via antiapoptotic [26], supportive (i.e., stimulation of mitosis, proliferation, and differentiation) [27], and angiogenic effects [26]. In our previous experimental studies, we have demonstrated the therapeutic potential of MSCs in animal models of parkinsonian disorders through anti-inflammatory action [28], autophagy modulation [29], stabilization of axonal transport [18], control of microglia M2 polarization [30], proteolysis of *α*-synuclein aggregates [17], and inhibition of *α*-synuclein transmission [16], which might modulate *α*-synuclein-related microenvironments. Furthermore, in two previous clinical trials [12, 13], we demonstrated the safety and clinical efficacy of intra-arterial and intravenous administration of autologous BM-MSCs in patients with MSA-C. However, our previous protocols were criticized for the barrier to the delivery of MSCs [14, 31]: although intravenous injection is the least invasive procedure, it may lead to the majority of MSCs being trapped in the lung, liver, and spleen [14, 32]. Intra-arterial administration allows a larger number of MSCs to reach the brain [32], although the BBB likely still comprises a major hurdle to MSC access [14]. For this reason, we slightly modified the protocol of BM-MSC administration (i.e., simply via intra-arterial routes) in the present trial. Additionally, we adjusted the dose of BM-MSCs based on a preclinical study and then estimated the maximum tolerated dose of BM-MSCs using a three-stage dose escalation schedule.

In the present study, we observed no SAEs corresponding to DLT. As a moderate level SUSAR, we saw one case of leptomeningeal enhancement in the cerebellar folia, along with enhancement in the right superior colliculus and right thalamus 1 day after the intra-arterial administration of MSCs. Clinically, these MRI findings might be attributed to BBB breakdown with increased permeability. We previously provided clinical and radiological evidence of increased BBB permeability in patients with MSA [33], although it remains unclear whether this was due to a compensatory response to the neurodegenerative process or as a direct consequence of disease progression. MSCs can also penetrate the BBB [34, 35] either through transiently formed interendothelial gaps [36] or via interaction with adhesion molecules and activation of matrix metalloproteinases [37]. Therefore, the early-stage patient with MS in the present study who showed leptomeningeal enhancement in the cerebellar folia likely had a more efficient delivery of MSCs to the brain through the porous BBB [38–40]. Additionally, the reversible hyperintensity in the right superior colliculus and thalamus may have been related to a reactive implantation response to MSCs. A similar abnormal MRI finding was observed in a recently reported clinical trial of intrathecal administration of autologous MSCs in patients with MSA [14]. Taken together, this case demonstrated that the BBB penetrance of intra-arterially administrated MSCs is not associated with serious adverse effects in patients with MSA. Furthermore, none of the patients from the present study showed acute cerebral ischemic lesions on brain MRI after the procedure in this study. In a previous study, 28.6% of MSC-treated patients had ischemic spots [13]. This discrepancy may be due to the small number of patients in the present trial; it is less likely that the spotty ischemic lesions on diffusion-weighted images were secondary to the MSCs themselves or related to the MSC concentrations used in the present trial.

The present study failed to reveal whether MSC treatment was associated with slower disease progression assessed using the UMSARS scores. However, it is not surprising that statistically significant results could not be obtained because the number of patients was small in each treatment group. In fact, there was a dose-dependent trend, with the medium- and high-dose groups tending to have a slower rate of increase in UMSARS scores than the low-dose group. Moreover, these negative results may have arisen because the observation periods were insufficient to investigate disease progression. In our previous study, a significant treatment effect was observed at day 240 after initial MSCs treatment, implying that there is a considerable time lag until the neuroprotective effect of MSCs is clinically evident [13]. Additional studies with a large sample size and high-dose MSC therapy are required to investigate the clinical efficacy of MSCs. Meanwhile, we found that the plasma levels of proinflammatory cytokines, including IL-1*β*, TNF-*α*, and MCP-1, tended to be decreased after MSC treatment in the medium- and high-dose groups. These findings suggest that MSCs have a neuroprotective effect in patients with MSA, although the clinical benefits and changes in plasma cytokine levels did not reach statistical significance.

Our study had some limitations. First, it was designed as a single-center study because the procedures such as harvest and intra-arterial administration of MSCs required skilled and experienced experts. Secondly, the number of study participants was too small to draw significant results. Additionally, a single injection of MSCs may not be sufficient to exert an effect [13], so the safety and optimal timing of repeated injections should be further investigated. The placebo effect of MSC therapy may occur in some patients, and caution should be exercised when interpreting the results. Furthermore, a 28-day follow-up period may be too short to determine the AEs of MSC therapy per se. In fact, eight participants visited the clinic for more than a year after MSC therapy, and additional 22 adverse events were reported. However, none of the AEs were considered ADR (Table S4). Thirdly, the study was confined to patients with MSA-C, whereas the European and North American MSA Study Groups reported a predominance of the MSA-parkinsonian (MSA-P) subtype over the MSA-C subtype [41, 42]. In fact, patients with MSA-P appear to have a distinct clinical course from patients with MSA-C [43, 44], and additional studies are required to validate the safety and tolerability of MSCs in patients with MSA-P. Fourthly, cerebrospinal fluid (CSF) analysis would be more relevant to elucidating mechanisms for possible neuroprotective effects of MSCs than blood sample analysis, and CSF sample collection should be considered in future studies.

## 5. Conclusions

In conclusion, our study confirmed the safety of a single intra-arterial administration of autologous BM-MSCs in patients with MSA-C, although the clinical efficacy and changes in plasma cytokine levels were not evident due to the small sample size. These findings suggest that MSC therapy via the intra-arterial route is a safe and feasible therapeutic strategy that can lead to phase II and III clinical trials in MSA-C in the future.

## Figures and Tables

**Table 1:** Demographic and clinical characteristics of study participants.

Case ID	Age (yr)	Sex	Age of	Disease	UMSARS at screening (V1)			
			Onset (yr)	Duration (mo)	Part I	Part II	Part IV	Total
Low-dose group								
S-101	62	F	58	39.0	19	24	3	46
S-102	49	M	46	33.3	21	20	2	43
S-103	64	F	61	30.1	20	17	3	40

Medium-dose group								
S-201	63	M	59	44.7	17	20	3	40
S-202	68	M	67	16.6	17	18	2	37
S-203	57	M	55	20.7	15	14	1	30

High-dose group								
S-301	52	M	51	21.0	18	22	2	42
S-302	51	F	49	32.5	24	17	3	44
S-303	48	F	47	11.1	14	17	2	33

UMSARS: Unified Multiple System Atrophy Rating Scale.

**Table 2:** Profiles of adverse events in study participants.

	Low-dose group		Medium-dose group		High-dose group		Total (*N* = 9)	
	*n* (%)	Events	*n* (%)	Events	*n* (%)	Events	*n* (%)	Events
Total adverse event	3 (100.0%)	13	0 (0.0%)	0	2 (66.7%)	2	5 (55.6%)	15
Nervous system disorders	2 (66.7%)	2	0 (0.0%)	0	1 (33.3%)	1	3 (33.3%)	3
Blood-brain barrier breakdown^a,b^	1 (33.3%)	1	0 (0.0%)	0	0 (0.0%)	0	1 (11.1%)	1
Headache	0 (0.0%)	0	0 (0.0%)	0	1 (33.3%)	1	1 (11.1%)	1
Photopsia	1 (33.3%)	1	0 (0.0%)	0	0 (0.0%)	0	1 (11.1%)	1
Gastrointestinal disorders	1 (33.3%)	2	0 (0.0%)	0	1 (33.3%)	1	2 (22.2%)	3
Constipation	1 (33.3%)	1	0 (0.0%)	0	1 (33.3%)	1	2 (22.2%)	2
Abdominal distension	1 (33.3%)	1	0 (0.0%)	0	0 (0.0%)	0	1 (11.1%)	1
Investigations	2 (66.7%)	2	0 (0.0%)	0	0 (0.0%)	0	2 (22.2%)	2
Blood pressure increased	1 (33.3%)	1	0 (0.0%)	0	0 (0.0%)	0	1 (11.1%)	1
Weight increased	1 (33.3%)	1	0 (0.0%)	0	0 (0.0%)	0	1 (11.1%)	1
Infections and infestations	1 (33.3%)	2	0 (0.0%)	0	0 (0.0%)	0	1 (11.1%)	2
Herpes zoster	1 (33.3%)	1	0 (0.0%)	0	0 (0.0%)	0	1 (11.1%)	1
Urinary tract infection^a^	1 (33.3%)	1	0 (0.0%)	0	0 (0.0%)	0	1 (11.1%)	1
Injury, poisoning and procedural complications	1 (33.3%)	2	0 (0.0%)	0	0 (0.0%)	0	1 (11.1%)	2
Fall	1 (33.3%)	1	0 (0.0%)	0	0 (0.0%)	0	1 (11.1%)	1
Rib fracture	1 (33.3%)	1	0 (0.0%)	0	0 (0.0%)	0	1 (11.11)	1
Psychiatric disorders	1 (33.3%)	2	0 (0.0%)	0	0 (0.0%)	0	1 (11.11)	2
Anxiety	1 (33.3%)	1	0 (0.0%)	0	0 (0.0%)	0	1 (11.11)	1
Depression	1 (33.3%)	1	0 (0.0%)	0	0 (0.0%)	0	1 (11.11)	1

All the terminologies of medical signs, symptoms, and disorders were coded as MedDRA (ICH, v22.1). ^a^Serious adverse event. ^b^Adverse drug reaction (i.e., adverse event attributable to the MSCs treatment).

**Table 3:** Longitudinal changes in UMSARS scores.

	Low-dose group	Medium- and high-dose groups	Overall *p* value	Post hoc *p* value^a^
UMSARS part I				
V1 (-1 month)	20.00 (1.74)	17.50 (1.23)	Group: 0.184	0.418
V7 (+1 month)	19.67 (2.05)	18.67 (1.45)	Time: 0.020	0.613
V8 (+3 months)	26.67 (3.13)	20.17 (2.21)	Group × time: 0.237	0.401
UMSARS part II				
V1 (-1 month)	20.33 (1.73)	18.00 (1.22)	Group: 0.165	0.458
V7 (+1 month)	20.33 (2.95)	17.50 (2.08)	Time: 0.049	0.458
V8 (+3 months)	25.33 (2.39)	18.33 (1.69)	Group × time: 0.131	0.144
Total UMSARS				
V1 (-1 month)	43.00 (2.79)	37.67 (1.97)	Group: 0.111	0.244
V7 (+1 month)	42.67 (4.46)	38.33 (3.16)	Time: 0.008	0.454
V8 (+3 months)	55.33 (5.14)	40.83 (3.64)	Group × time: 0.096	0.165

Values are expressed as estimated mean (standard error). Abbreviations: UMSARS: Unified Multiple System Atrophy Rating Scale; MSC: mesenchymal stem cell. ^a^False discovery rate (FDR) adjusted *p* value.

## Data Availability

For purposes of replicating procedures and results, any qualified investigator can request anonymized data after ethics clearance and approval by all authors.

## Supplementary Materials

Supplementary Materials Supplementary Methods, 4 Supplementary Tables, and 1 Supplementary Figure. Table S1: flow cytometry findings of harvested mesenchymal stem cells in study participants. Table S2: longitudinal changes in UMSRAS scores of study participants. Table S3: longitudinal changes in cytokines and neurofilament light chain. Table S4: profiles of adverse events in study participants (1~12 months after mesenchymal stem cell therapy). Figure S1: changes in the plasma level of cytokines and neurofilament light chain throughout the follow-up period.
