# Supplementary material for: Phase I Trial of Intra-arterial Administration of Autologous Bone Marrow-Derived Mesenchymal Stem Cells in Patients with Multiple System Atrophy
Source: Stem Cells Int. 2021 Oct 19;2021:9886877. doi: 10.1155/2021/9886877 (PMC8548132; doi:10.1155/2021/9886877)

**Supplementary Methods**

**Preclinical study for dose selection**

Single-dose toxicity study was conducted at non-clinical Contract Research Organization, Good Laboratory Practice certified facility (ChemOn Inc., South Korea). The dose-dependent toxicity of CS10BR05 was evaluated using Sprague-Dawley Rats with a single administration of 1.5 × 10^5^ cells/head, 1.5 × 10^6^ cells/head, and 7.5 × 10^6^ cells/head through internal carotid arterial injection. The non-observed-adverse-effect level (NOAEL) was determined at 1.5 × 10^5^ cells/head. Considering the body weight of the rat, the clinical dose of CS10BR05 was set at 6.0 × 10^5^ cells/kg based on the NOAEL of the preclinical data.

**Cytokine Bio-Plex Assay**

The plasma levels of 27 cytokines (interleukin [IL]-1β, IL-1Ra, IL-2, IL-4, IL-5, IL-6, IL-7, IL-8, IL-9, IL-10, IL-12 (p70), IL-13, IL-15, IL-17A, eotaxin, basic fibroblast growth factor [bFGF], granulocyte colony stimulating factor [G-CSF], granulocyte/macrophagy colony stimulating factor [GM-CSF], interferon-γ [IFN-γ], IFN-γ-induced protein-10 [IP-10], monocyte chemoattractant protein-1 [MCP-1], macrophage inflammatory protein [MIP]-1α, MIP-1β, platelet-derived growth factor-BB [PDGF-BB], C-C motif chemokine ligand 5 [CCL5], tumor necrosis factor-α [TNF-α], and vascular endothelial growth factor [VEGF]) were measured using the Bio-Plex200 Cytokine Assay System (Bio-Rad Laboratories, Hercules, CA, USA). We selected these cytokines according to their roles as follows: (1) IL-1β, IL-1Ra, IL-6, IL-7, and TNF-α, are pleiotropic cytokines with various functions such as pro-inflammation, anti-inflammation, apoptosis, and immune modulation; (2) IL-2, IL-12 (p70), IL-15, and IFN-γ are Th1-related cytokines; (3) IL-4, IL-5, IL-9, IL-10, and IL-13 are Th2-related cytokines; (4) IL-17A is a Th17-related cytokine that can induce the expression of pro-inflammatory cytokines and chemokines; (5) IL-8, eotaxin, IP-10, MCP-1, MIP-1α, MIP-1β, and CCL5 are related to inflammatory responses and promote the migration of immune cells; and (6) bFGF, G-CSF, GM-CSF, PDFG-BB, and VEGF are growth factors and cytokines related to hematopoiesis and angiogenesis. The calibration curve for each cytokine was analyzed using the five-parameter logistic curve regression in Bio-Plex manager 6.1 software (Bio-Rad Laboratories, Hercules, CA, USA). Standard values were considered acceptable if the point fell within 70−130% of the expected values as recommended by the manufacturer.

**Neurofilament light chain immunoassay**

Changes in plasma level of neurofilament light chain (NF-L) were assessed using enzyme-linked immunosorbent assay (ELISA) (ready-to-use sandwich ELISA kit [LS-F6701]; LSBio, Seattle, WA). Standard and plasma samples were added to the anti-NF-L capture antibody-precoated wells and incubated for 1 hour at 37°C. Next, biotinylated detection antibody reactions were conducted. The biotinylated anti-NF-L antibodies were detected by sequential incubation with a streptavidin-horseradish peroxidase conjugated and 3, 3’, 5, 5’-tetramethylbenzidine substrates. After a 10 to 20-minute colorimetric developing reaction, the optical density of the well was measured at a wavelength of 450 nm using a microplate reader (xMark; Bio-Rad).

**TABLE S1. Flow cytometry findings of harvested mesenchymal stem cells in study participants**

| Case ID | No. of isolated  NCs (× 10^6^ cells) | No. of injected MSCs (×10^6^ cells) | CD34 (%) | CD45 (%) | CD29 (%) | CD44 (%) | CD73 (%) | CD90 (%) | CD105 (%) |
| --- | --- | --- | --- | --- | --- | --- | --- | --- | --- |
| Low-dose group |  |  |  |  |  |  |  |  |  |
| S-101 | 521.000 | 61.933 | 0.23 | 0.13 | 99.93 | 99.87 | 99.80 | 99.97 | 90.80 |
| S-102 | 240.000 | 63.200 | 0.17 | 0.00 | 99.77 | 97.17 | 94.07 | 100.00 | 99.83 |
| S-103 | 460.333 | 188.000 | 0.13 | 0.03 | 99.67 | 99.17 | 99.47 | 98.80 | 95.10 |
| Medium-dose group |  |  |  |  |  |  |  |  |  |
| S-201 | 523.667 | 125.667 | 0.53 | 0.33 | 99.80 | 99.23 | 98.60 | 100.00 | 99.07 |
| S-202 | 326.333 | 167.667 | 0.23 | 0.13 | 99.77 | 99.77 | 98.87 | 99.93 | 96.73 |
| S-203 | 516.667 | 148.000 | 2.87 | 1.43 | 100.00 | 99.80 | 99.37 | 99.97 | 99.53 |
| High-dose group |  |  |  |  |  |  |  |  |  |
| S-301 | 469.000 | 216.333 | 0.00 | 0.17 | 99.43 | 99.80 | 98.93 | 99.93 | 93.97 |
| S-302 | 244.667 | 156.333 | 0.43 | 0.40 | 99.30 | 98.53 | 99.90 | 99.93 | 99.93 |
| S-303 | 697.334 | 151.667 | 0.03 | 0.40 | 99.80 | 99.77 | 96.40 | 99.90 | 95.43 |

Abbreviations: NC, mononuclear cells; MSCs, mesenchymal stem cells.

**TABLE S2. Longitudinal changes in UMSRAS scores of study participants**

| Case ID | UMSARS Part I | | |  | UMSARS Part II | | |  | Total UMSARS | | |
| --- | --- | --- | --- | --- | --- | --- | --- | --- | --- | --- | --- |
|  | V1 | V7 | V8 |  | V1 | V7 | V8 |  | V1 | V7 | V8 |
| Low-dose group |  |  |  |  |  |  |  |  |  |  |  |
| S-101 | 19 | 18 | 32 |  | 24 | 19 | 28 |  | 46 | 40 | 64 |
| S-102 | 21 | 21 | 30 |  | 20 | 22 | 24 |  | 43 | 45 | 57 |
| S-103 | 20 | 20 | 18 |  | 17 | 20 | 24 |  | 40 | 43 | 45 |
| Medium-dose group |  |  |  |  |  |  |  |  |  |  |  |
| S-201 | 17 | 14 | 15 |  | 20 | 17 | 17 |  | 40 | 34 | 34 |
| S-202 | 17 | 21 | 22 |  | 18 | 18 | 22 |  | 37 | 41 | 47 |
| S-203 | 15 | 13 | 16 |  | 14 | 13 | 16 |  | 30 | 27 | 33 |
| High-dose group |  |  |  |  |  |  |  |  |  |  |  |
| S-301 | 18 | 22 | 26 |  | 22 | 29 | 26 |  | 42 | 54 | 55 |
| S-302 | 24 | 22 | 23 |  | 17 | 14 | 14 |  | 44 | 38 | 40 |
| S-303 | 14 | 20 | 19 |  | 17 | 14 | 15 |  | 33 | 36 | 36 |

V1, V7, V8 = 1 month before, 1 month after, and 3 months after intra-arterial MSCs treatment.

**TABLE S3. Longitudinal changes in cytokines and neurofilament light chain**

| Case ID | IL-1β | | |  | TNF-α | | |  | MCP-1 | | |  | NF-L | | |
| --- | --- | --- | --- | --- | --- | --- | --- | --- | --- | --- | --- | --- | --- | --- | --- |
|  | V4 | V6 | V7 |  | V4 | V6 | V7 |  | V4 | V6 | V7 |  | V4 | V6 | V7 |
| Low-dose group |  |  |  |  |  |  |  |  |  |  |  |  |  |  |  |
| S-101 | 17.80 | 19.63 | 31.47 |  | 45.50 | 34.83 | 41.00 |  | 128.20 | 97.20 | 99.37 |  | 396.47 | 448.55 | 330.64 |
| S-102 | 31.30 | 59.63 | 29.80 |  | 28.50 | 122.50 | 45.67 |  | 68.70 | 134.87 | 62.53 |  | 196.28 | 244.12 | 283.31 |
| S-103 | 11.47 | 13.30 | 21.47 |  | 31.67 | 32.17 | 41.83 |  | 72.53 | 65.37 | 61.53 |  | 170.32 | 265.33 | 300.95 |
| Medium-dose group |  |  |  |  |  |  |  |  |  |  |  |  |  |  |  |
| S-201 | 59.87 | 28.53 | 28.03 |  | 34.53 | 29.37 | 21.03 |  | 85.60 | 88.50 | 75.67 |  | 184.57 | 241.41 | 273.64 |
| S-202 | 33.70 | 15.03 | 11.37 |  | 71.53 | 29.53 | 27.03 |  | 107.33 | 89.33 | 90.50 |  | 387.14 | 389.51 | 380.35 |
| S-203 | 10.20 | 20.70 | 20.37 |  | 19.87 | 34.20 | 22.03 |  | 26.33 | 39.50 | 36.00 |  | 151.32 | 194.24 | 150.64 |
| High-dose group |  |  |  |  |  |  |  |  |  |  |  |  |  |  |  |
| S-301 | 18.03 | 11.37 | 10.70 |  | 24.03 | 18.37 | 26.37 |  | 84.67 | 73.83 | 58.17 |  | 382.22 | 307.91 | 345.47 |
| S-302 | 6.03 | 25.53 | 20.70 |  | 18.53 | 50.37 | 48.20 |  | 49.33 | 69.83 | 71.67 |  | 190.68 | 182.20 | 183.05 |
| S-303 | 9.20 | 2.03 | 2.70 |  | 29.03 | 11.70 | 11.53 |  | 63.33 | 48.33 | 40.67 |  | 197.47 | 168.63 | 190.85 |

V4, V6, V7 = 0 day, 14 day, and 28 day after intra-arterial MSCs treatment.

**TABLE S4. Profiles of adverse events in study participants (1~12 months after mesenchymal stem cell therapy)**

|  | Low-dose group | |  | Medium-dose group | |  | High-dose group | |  | Total (N = 8) | |
| --- | --- | --- | --- | --- | --- | --- | --- | --- | --- | --- | --- |
|  | n (%) | events |  | n (%) | events |  | n (%) | events |  | n (%) | events |
| Total Adverse Event | 2 (100.0%) | 10 |  | 3 (100.0%) | 5 |  | 3 (100.0%) | 7 |  | 8 (100.0%) | 22 |
| Nervous system disorders | 1 (50.0%) | 1 |  | 0 (0.0%) | 0 |  | 0 (0.0%) | 0 |  | 1 (12.5%) | 1 |
| Syncope | 1 (50.0%) | 1 |  | 0 (0.0%) | 0 |  | 0 (0.0%) | 0 |  | 1 (12.5%) | 1 |
| Gastrointestinal disorders | 1 (50.0%) | 2 |  | 0 (0.0%) | 0 |  | 0 (0.0%) | 1 |  | 1 (12.5%) | 2 |
| Constipation | 1 (50.0%) | 1 |  | 0 (0.0%) | 0 |  | 0 (0.0%) | 1 |  | 1 (12.5%) | 1 |
| Dry mouth | 1 (50.0%) | 1 |  | 0 (0.0%) | 0 |  | 0 (0.0%) | 0 |  | 1 (12.5%) | 1 |
| General disorder & administration site condition | 1 (50.0%) | 1 |  | 1 (33.3%) | 1 |  | 0 (0.0%) | 0 |  | 2 (25.0%) | 2 |
| Pain | 1 (50.0%) | 1 |  | 0 (0.0%) | 0 |  | 0 (0.0%) | 0 |  | 1 (12.5%) | 1 |
| Pyrexia | 0 (0.0%) | 0 |  | 1 (33.3%) | 1 |  | 0 (0.0%) | 0 |  | 1 (12.5%) | 1 |
| Investigations | 1 (50.0%) | 1 |  | 1 (33.3%) | 1 |  | 2 (66.6%) | 2 |  | 4 (50.0%) | 5 |
| Sputum abnormal | 1 (50.0%) | 1 |  | 0 (0.0%) | 0 |  | 1 (33.3%) | 1 |  | 1 (12.5%) | 1 |
| Weight decreased | 1 (50.0%) | 1 |  | 0 (0.0%) | 0 |  | 0 (0.0%) | 0 |  | 2 (25.0%) | 2 |
| ALT/AST increased | 0 (0.0%) | 0 |  | 1 (33.3%) | 1 |  | 0 (0.0%) | 0 |  | 1 (12.5%) | 1 |
| Blood urine | 0 (0.0%) | 0 |  | 0 (0.0%) | 0 |  | 1 (33.3%) | 1 |  | 1 (12.5%) | 1 |
| Infections and infestations | 1 (50.0%) | 1 |  | 1 (33.3%) | 1 |  | 1 (33.3%) | 1 |  | 3 (37.5%) | 3 |
| Pneumonia ^a^ | 0 (0.0%) | 0 |  | 1 (33.3%) | 1 |  | 0 (0.0%) | 0 |  | 1 (12.5%) | 1 |
| Urinary tract infection | 1 (50.0%) | 1 |  | 0 (0.0%) | 0 |  | 1 (33.3%) | 1 |  | 2 (25.0%) | 2 |
| Injury, poisoning and procedural complications | 0 (0.0%) | 0 |  | 1 (33.3%) | 1 |  | 1 (33.3%) | 1 |  | 2 (25.0%) | 2 |
| Fall | 0 (0.0%) | 0 |  | 0 (0.0%) | 0 |  | 1 (33.3%) | 1 |  | 1 (12.5%) | 1 |
| Skin laceration | 0 (0.0%) | 0 |  | 1 (33.3%) | 1 |  | 0 (0.0%) | 0 |  | 1 (12.5%) | 1 |
| Psychiatric disorders | 2 (100.0%) | 2 |  | 0 (0.0%) | 0 |  | 0 (0.0%) | 0 |  | 2 (25.0%) | 2 |
| Insomnia | 1 (50.0%) | 1 |  | 0 (0.0%) | 0 |  | 0 (0.0%) | 0 |  | 1 (12.5%) | 1 |
| Depression | 1 (50.0%) | 1 |  | 0 (0.0%) | 0 |  | 0 (0.0%) | 0 |  | 1 (12.5%) | 1 |
| Skin and subcutaneous tissue disorders | 1 (50.0%) | 1 |  | 0 (0.0%) | 0 |  | 1 (33.3%) | 1 |  | 2 (25.0%) | 2 |
| Rash | 1 (50.0%) | 1 |  | 0 (0.0%) | 0 |  | 1 (33.3%) | 1 |  | 2 (25.0%) | 2 |
| Renal and urinary disorders | 0 (0.0%) | 0 |  | 0 (0.0%) | 0 |  | 1 (33.3%) | 1 |  | 1 (12.5%) | 1 |
| Chronic kidney disease | 0 (0.0%) | 0 |  | 0 (0.0%) | 0 |  | 1 (33.3%) | 1 |  | 1 (12.5%) | 1 |
| Surgical and medical procedures | 0 (0.0%) | 0 |  | 1 (33.3%) | 1 |  | 0 (0.0%) | 0 |  | 1 (12.5%) | 1 |
| Tooth extraction | 0 (0.0%) | 0 |  | 1 (33.3%) | 1 |  | 0 (0.0%) | 0 |  | 1 (12.5%) | 1 |
| Eye disorders | 0 (0.0%) | 0 |  | 0 (0.0%) | 0 |  | 1 (33.3%) | 1 |  | 1 (12.5%) | 1 |
| Conjunctival disorder | 0 (0.0%) | 0 |  | 0 (0.0%) | 0 |  | 0 (0.0%) | 0 |  | 1 (12.5%) | 1 |

Of the nine participants in this study, eight patients (two in the low-dose group, three in the medium-dose group, and three in the high-dose group) were followed up for at least one year. One patient in the low-dose group was followed up for 3 months. During the follow-up period up to 1 year, additional 22 adverse events were reported in 8 patients with MSA-C. None of the adverse events were considered adverse drug reaction. All the terminology of medical signs, symptoms, and disorders were coded as MedDRA (ICH, v24.0).

^a^ Serious Adverse Event

**FIGURE S1: Changes in the plasma level of cytokines and neurofilament light chain throughout the follow-up period.** Low-dose group (n = 3) versus Medium- and High-dose groups (n = 6). **(A) Interleukin-1β** (IL-1 β). **(B) Tumor necrosis factor-α** (TNF- α). **(C) Monocyte chemoattractant protein-1** (MCP-1). **(D) Neurofilament light chain** (NF-L). There was a trend toward decreases in the concentrations of pro-inflammatory cytokines, including IL-1β (*p* = 0.380), TNF-α (*p* = 0.438), and MCP-1 (*p* = 0.199) in the medium- and high-dose groups, although these changes did not reach statistical significance.


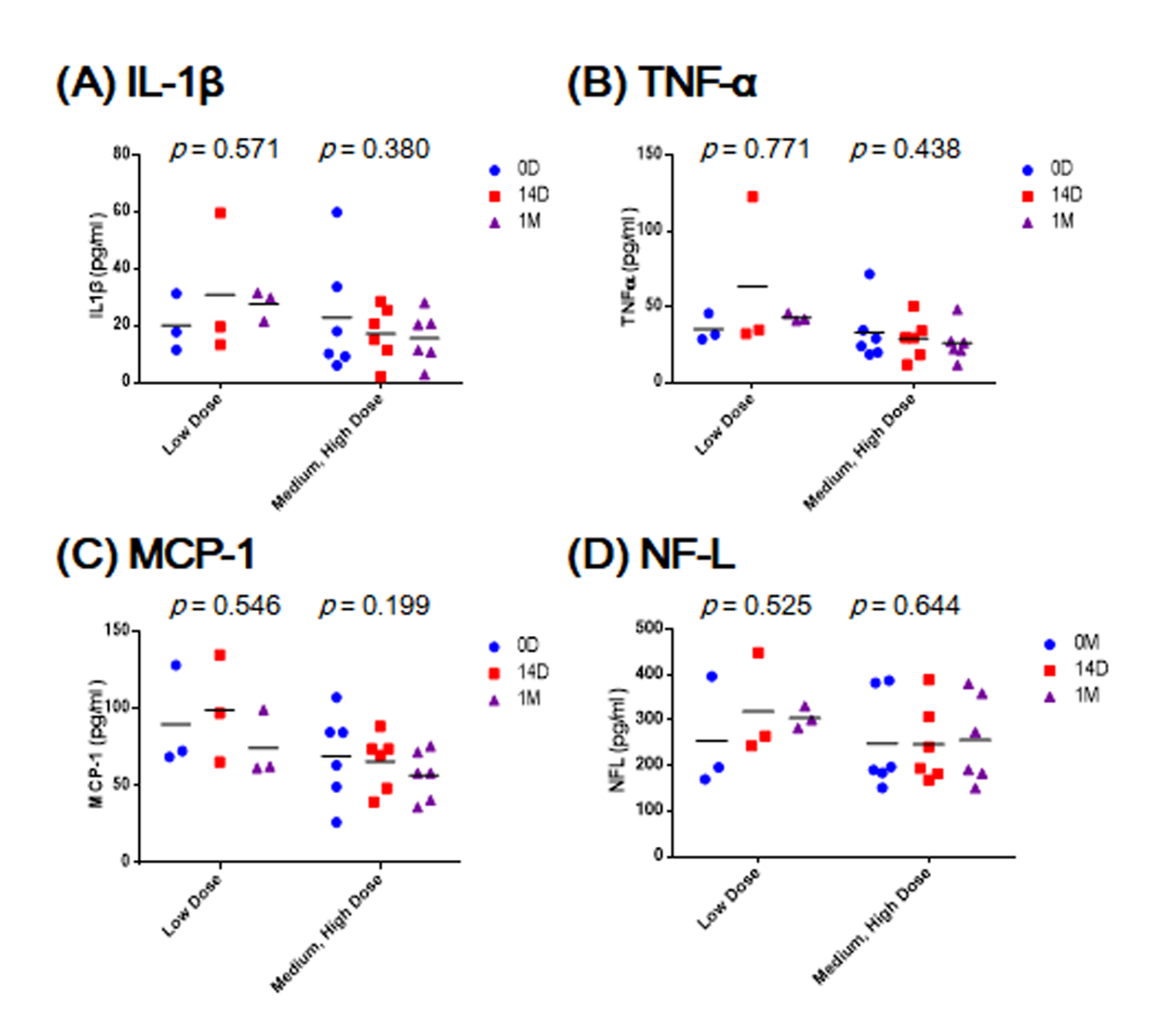

Supplement: Supplementary Materials — Supplementary Methods, 4 Supplementary Tables, and 1 Supplementary Figure. Table S1: flow cytometry findings of harvested mesenchymal stem cells in study participants. Table S2: longitudinal changes in UMSRAS scores of study participants. Table S3: longitudinal changes in cytokines and neurofilament light chain. Table S4: profiles of adverse events in study participants (1~12 months after mesenchymal stem cell therapy). Figure S1: changes in the plasma level of cytokines and neurofilament light chain throughout the follow-up period. [file 9886877.f1.docx]
